# Epidemiology, Pathogenesis, Clinical Features, and Management of Non-HACEK Gram-Negative Infective Endocarditis

**DOI:** 10.3390/antibiotics14100980

**Published:** 2025-09-29

**Authors:** Roberta Monardo, Rebecka Papaioannu Borjesson, Giacomo Ponta, Antonella Castagna, Marco Ripa

**Affiliations:** 1 Unit of Infectious Diseases, Vita-Salute San Raffaele University, 20132 Milan, Italy; 2 Unit of Infectious Diseases, IRCCS San Raffaele Scientific Institute, 20127 Milan, Italy

**Keywords:** Infective Endocarditis, gram-negative, *Pseudomonas aeruginosa*, *Serratia marcescens*, *Klebsiella pneumoniae*, *Escherichia coli*

## Abstract

**Background/Objectives:** Non-HACEK Gram-Negative Infective Endocarditis (NHGNIE) is a rare but increasingly recognized condition associated with high morbidity and mortality. Its incidence is rising among people who inject drugs (PWID), patients with prosthetic valves or cardiac devices, and those with significant healthcare exposure. We aimed to provide a comprehensive review of the epidemiology, pathogenesis, diagnosis, clinical features, and management of NHGNIE. **Methods:** We conducted a narrative synthesis of published cohort studies, case series, guideline documents, and recent registry data addressing NHGNIE. Evidence was extracted and critically appraised with emphasis on epidemiological patterns, microbial etiology, diagnostic frameworks, therapeutic strategies, and outcomes. Special focus was given to pathogen-specific differences and the impact of antimicrobial resistance. **Results:** NHGNIE accounts for approximately 1.5–10.7% of IE cases worldwide, with marked geographical variability. *Pseudomonas aeruginosa*, *Serratia marcescens*, *Klebsiella pneumoniae*, and *Escherichia coli* are the predominant pathogens, with clinical profiles differing between younger, PWID-based populations and older, comorbidity-affected cohorts. Advances in molecular diagnostics and imaging have improved case identification, though pathogen-specific diagnostic performance remains limited. Outcomes are poor, with in-hospital mortality up to 41%. Antimicrobial therapy is complicated by biofilm formation and potential for multidrug resistance; evidence for combination therapy versus monotherapy is conflicting. Surgical intervention appears to improve outcomes when performed according to guideline-based indications, but results are heterogeneous across studies. **Conclusions:** NHGNIE is a clinically significant form of IE with complex epidemiology, diagnostic challenges, and limited evidence to guide treatment. Effective management requires individualized care coordinated within a multidisciplinary “endocarditis team”.

## 1. Introduction

Non-HACEK (*Haemophilus*, *Aggregatibacter*, *Cardiobacterium*, *Eikenella*, *Kingella*) Gram-Negative Infective Endocarditis (NHGNIE) represents a rare but increasingly recognized clinical entity that poses significant diagnostic and therapeutic challenges. Traditionally considered an uncommon cause of IE, NHGNIE is now being reported with greater frequency, particularly among populations at risk, such as people who inject drugs, patients with prosthetic valves or cardiac devices, and individuals with substantial healthcare exposure. The clinical relevance of NHGNIE stems not only from its growing incidence but also from its association with multidrug-resistant organisms, complicated clinical courses, and suboptimal outcomes compared with other etiologies of IE. This review provides a comprehensive overview of the epidemiology, pathogenesis, diagnostic advances, clinical manifestations, therapeutic strategies, and future research needs surrounding NHGNIE.

## 2. Epidemiology and Etiology

**Figure 1:**
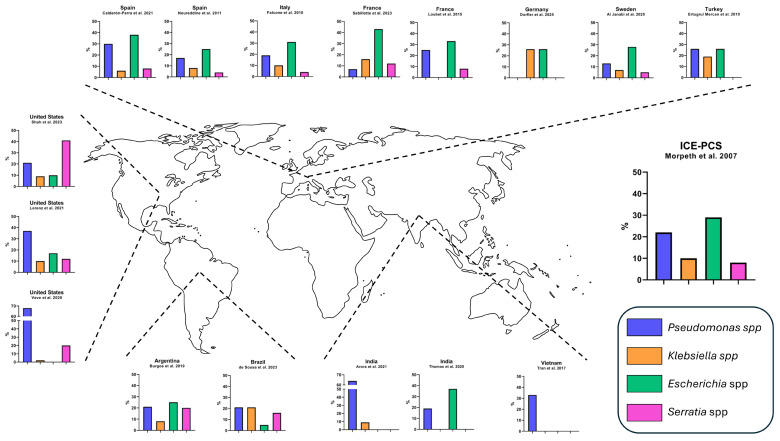
Microbial etiology according to geographical distribution. References: [1,2,3,4,5,6,7,8,9,10,11,12,13,14,15,16,17].

NHGNIE is a rare but increasingly recognized clinical entity. Although it accounts for a small proportion of all IE cases, its incidence appears to be relevant, particularly among people who inject drugs (PWID) and individuals with significant comorbidities or healthcare exposure. The epidemiological characteristics of selected cohorts of patients with NHGNIE are described in Table 1. Microbial etiology according to geographical distribution is illustrated in Figure 1 and described in the Supplementary Materials, Table S1.

A U.S.-based study [18] estimated that the risk of IE in patients with Gram-negative bacteremia is approximately 14%. Notably, in individuals with cardiac devices, *Serratia marcescens* and *Pseudomonas aeruginosa* (alongside *Staphylococcus aureus*) were significantly associated with an elevated risk of device-related infection compared to other pathogens [19]. As a result, these organisms have been recognized as typical causative agents in the updated DUKE-ISCVID diagnostic criteria for IE [20].

Large international and national registries have reported NHGNIE in approximately 1.5–4% of cases [1,5,7,8,9,10,11,12,14,17]. Higher proportions have been observed in regions where PWID or healthcare-associated infections are common, reaching 5–10% in several cohorts from the United States and India [3,15,16]. By contrast, single-center European studies often describe lower frequencies, typically in the 1–3% range [7,8,9,11,12,13,14].

The distribution of pathogens varies according to geographic and demographic contexts. Across all cohorts, the most common agents are *P. aeruginosa*, *Escherichia coli*, *Klebsiella pneumoniae*, and *S. marcescens* [1,2,3,4,5,6,7,8,9,10,11,12,13,14,15,16,17,21]. In studies dominated by PWID, particularly in the United States and India, *P. aeruginosa* and *Serratia* spp. predominate [2,3,4,15]. In contrast, European and South American series, which largely involve older patients with healthcare exposure, most frequently report *E. coli* and *Klebsiella* spp. as leading etiologies [5,6,7,9,11,12,14]. This variation likely reflects local epidemiology, with injection drug use driving *Pseudomonas* and *Serratia* infections and nosocomial or urinary tract sources favoring *E. coli* and *Klebsiella* spp.

Two distinct clinical profiles can therefore be recognized. In PWID-associated cohorts, patients are generally younger and frequently have a history of prior IE. Infections in this population often involve native or right-sided valves and are predominantly caused by *P. aeruginosa* and *S. marcescens* [2,3,4,15]. In contrast, healthcare-associated or comorbidity-driven NHGNIE typically affects older patients, with a high burden of comorbidities (such as diabetes, malignancy, chronic kidney disease, or immunosuppression), and acquisition is frequently nosocomial, linked to urinary or gastrointestinal sources or to indwelling central venous catheters. In these settings, *E. coli* and *Klebsiella* spp. are the predominant pathogens [5,6,7,8,9,10,11,12,14].

The frequency of prosthetic valve endocarditis (PVE) among patients with NHGNIE also differs between settings. In comorbidity-heavy, healthcare-associated cohorts, PVE is common, with reported rates ranging from 25% to more than 60% [5,6,7,9,10,11,12,14]. In contrast, among PWID-dominated populations, native valve involvement is more common, and PVE is observed much less frequently, typically below 30% [2,3,4,15].

Additional epidemiological observations include sex- and valve-specific trends. Interestingly, one study from Israel [22] found a higher frequency of Gram-negative IE among female patients undergoing cardiac surgery (20% vs. 11%). Moreover, some cohorts have reported a disproportionate association of Gram-negative pathogens with right-sided IE [23]. These associations, however, are not consistent across studies and likely reflect local epidemiological differences.

The rising prevalence of multidrug-resistant (MDR) organisms [24] raises concern that MDR-NHGNIE will play an expanding role in future clinical practice [25]. Notably, a study from Romania [26] found that 86% of Gram-negative isolates were classified as MDR, with resistance to three or more drug classes in 38% of cases, in a population with a high burden of comorbidities and health exposure. Similarly, a study from India [16], reported resistance to cephalosporins in 55% of cases and to carbapenems in 11%. In the largest available cohort of patients with NHGNIE [2], coming from the U.S., MDR prevalence was 21%, consistent with reports from other countries [3,5,13,14], but higher than those reported in France (10%), Germany (5%), and in another U.S. study (7%) [4,7,9].

In summary, NHGNIE remains a relatively rare but clinically significant entity, accounting for approximately 1.5–10.7% of IE cases across various cohorts. The epidemiology differs markedly according to underlying risk factors. PWID-associated cohorts are characterized by younger patients with native or right-sided IE, predominantly caused by *P. aeruginosa* or *Serratia* spp. Healthcare-associated cohorts involve older, comorbid patients, most often with urinary or catheter-related sources, and are usually due to *E. coli* or *Klebsiella* spp., with PVE being more frequently observed. Against this background, the increasing prevalence of MDR organisms underscore NHGNIE as an evolving clinical challenge.

## 3. Pathogenesis

### 3.1. Adhesion and Biofilm Formation in Gram-Negative Bacteria

The low incidence of IE caused by non-HACEK Gram-negative bacteria is largely due to their unique structural and functional features. Unlike many Gram-positive pathogens, most Gram-negative bacteria lack a protective capsule, making them more susceptible to innate immune defenses, especially the complement system. Furthermore, they typically do not express surface proteins that facilitate adhesion to extracellular matrix components or prosthetic materials, which are key steps in the pathogenesis of IE [27].

Experimental studies have shown that Gram-negative bacteria generally require a significantly higher infectious dose than Gram-positive ones to initiate IE, suggesting a lower intrinsic colonizing capacity [28]. In particular, non-HACEK strains often demonstrate a poor ability to adhere to cardiac endothelium and to establish biofilms, both of which are essential processes in the development of endocardial infection. An exception is *Salmonella* spp., which exhibits an enhanced ability to produce biofilm and colonize damaged cardiac tissues [1,28].

In the presence of predisposing conditions, such as valvular disease or the presence of prosthetic devices, some non-HACEK Gram-negative strains may overcome their limitations, leading to IE by enhancing survival in the endocardial vegetation [29]. However, these cases remain rare due to serum sensitivity and weak endothelial adhesion properties [28].

Biofilm development represents a key adaptive strategy that allows bacteria to withstand both antimicrobial treatments and host immune defenses [30,31]. These structures consist of communities of microorganisms enclosed in a matrix of extracellular polymeric substances (EPS) they produce themselves (primarily polysaccharides, proteins, and extracellular DNA) which promote adherence to surfaces and maintain the biofilm’s architecture [32,33].

Beyond serving as a structural framework, the EPS matrix limits the diffusion of antibiotics and immune effectors into the biofilm. Microorganisms residing within these communities display phenotypic traits that are distinct from their free-floating (planktonic) forms, including slowed metabolic activity and heightened resilience to nutrient deprivation, oxidative damage, and host immune responses. This protective environment can render bacteria 10 to 100 times less susceptible to antimicrobials, due to the combined effects of the physical matrix barrier and adaptive metabolic changes [30,31,34,35].

Within biofilms, bacteria engage in cell–cell communication through quorum sensing mechanisms, and the close proximity of cells facilitates the exchange of genetic material, enhancing adaptability and genomic diversity [30,32]. The structural organization of these communities is highly responsive to environmental cues, ranging from simple, flat layers to intricate three-dimensional formations such as those observed in *P. aeruginosa*. When nutrients become scarce, the development of microcolonies is often triggered, underscoring the dynamic and adaptive nature of biofilm growth [30].

The release of cells from established biofilms is a key step for spreading to new niches. This detachment may occur passively, through external physical factors, or actively, via controlled physiological processes regulated by the bacteria themselves [30].

The ability to form biofilms combined with antibiotic resistance constitutes a critical mechanism by which Gram-negative bacteria, despite species-specific variations, persist, colonize, and cause challenging infections, underscoring the need for targeted therapeutic and preventive strategies. Selected pathogenetic features of NHGN are described in Table 2.

### 3.2. Pseudomonas aeruginosa

*P. aeruginosa* is a significant Gram-negative pathogen in clinical contexts, recognized for its capacity to develop intricate and robust biofilm communities. These multicellular structures are key to its survival and persistence in both medical and natural environments, offering defense against host immune attacks and antibiotic treatments [36,37]. Relative to members of the *Enterobacterales* order, *P. aeruginosa* is known to develop more robust and extensive biofilms, highlighting its importance in clinical infections [38].

*P. aeruginosa* adheres to abiotic and biological surfaces through coordinated actions of type IV pili, flagella, and outer membrane adhesins, engaging surface-specific molecular targets and rapidly adapting its physiology to promote persistent colonization and biofilm formation [39,40,41].

The formation of *P. aeruginosa* biofilms involves several distinct phases: beginning with the attachment of planktonic bacteria to a surface, followed by growth and clustering into microcolonies, and finally reaching maturation characterized by the production of an extracellular matrix [42]. *P. aeruginosa* biofilms typically exhibit two main structural forms: a smooth, continuous layer and a complex three-dimensional configuration featuring tower- and mushroom-like structures connected by channels that enable the flow of nutrients and the removal of waste products [36,43].

The formation and regulation of these biofilms are controlled by multiple molecular pathways, with quorum sensing (QS) playing a pivotal role. The las and rhl QS systems regulate the expression of virulence factors and genes essential for proper biofilm development and maturation [44]. Strains lacking functional QS systems tend to form biofilms that are flatter, less complex in structure, and exhibit reduced adhesion to surfaces compared to their wild-type counterparts [45]. Apart from QS, additional elements like alginate and rhamnolipids play key roles in shaping the three-dimensional structure and enhancing the durability of biofilms, especially under stressful conditions [36,45]. Specifically, alginate synthesis boosts bacterial adhesion to surfaces and is markedly upregulated during chronic infections, for example in patients with cystic fibrosis [37].

Environmental factors play a significant role in shaping biofilm development. Elements such as nutrient concentration, available carbon sources, and oxygen levels can affect the biofilm’s architecture, progression, and eventual dispersal. Elevated nutrient conditions often induce biofilm dispersion, which correlates with increased expression of genes involved in flagellar motility, pili assembly, and anaerobic metabolic pathways. These dispersal strategies highlight the bacterium’s ability to adjust to fluctuating environmental conditions. The global regulatory gene *crc* is critical for carbon metabolism and biofilm development; its disruption leads to significant biofilm formation defects. Within the inner layers of biofilms, oxygen scarcity creates a challenging environment for survival. *P. aeruginosa* adapts by inducing genes involved in anaerobic respiration (including nir, nos, and nor clusters), which grants metabolic versatility under low-oxygen conditions. This ability supports its continued survival in hypoxic tissue regions [42].

The dispersal stage of biofilms involves significant changes in structure and cell behavior. Cells that detach from the biofilm exhibit different gene expression patterns and tend to be more sensitive to antibiotics than those that remain attached, with these modifications controlled through both transcriptional and post-translational mechanisms [46].

Moreover, *P. aeruginosa* secretes multiple virulence determinants such as exotoxin A, elastases, alkaline protease, and hemolytic phospholipase C, all of which play roles in damaging host tissues and impairing immune defenses [38].

The complex interplay of genetic, metabolic, and environmental factors controlling biofilm formation makes *P. aeruginosa* both a valuable model for biofilm research and a challenging pathogen in persistent infections, especially those involving indwelling medical devices.

### 3.3. Serratia marcescens

*S. marcescens* is an opportunistic Gram-negative bacterium increasingly acknowledged for its robust capacity to form biofilms, a characteristic intimately associated with its pathogenicity. The biosurfactant serrawettin is essential in enhancing surface motility and facilitating the initial phases of biofilm development [47,48]. Surface adhesins, particularly type I fimbriae encoded by genes such as fimA and fimC, play a critical role in epithelial cell attachment and the development of mature biofilms [49]. Additionally, factors like increased cell surface hydrophobicity and the synthesis of EPS contribute to stronger adhesion on medical devices, including prosthetic heart valves and catheters [34,50].

QS plays a key role in regulating processes such as surface adhesion, motility, biofilm development, and dispersal [49,51]. Biofilms formed by *S. marcescens* display a porous and filamentous architecture made up of interconnected chains and clusters of cells. Their development proceeds through a well-defined sequence that is largely controlled by QS mechanisms. Studies on QS-deficient mutants, which produce flat and disorganized biofilms, underscore the essential role of QS in establishing the typical three-dimensional structure [30,52].

Biofilm structure is also significantly affected by environmental conditions, with nutrient availability being a key determinant. In nutrient-rich environments, biofilms tend to develop filamentous forms, while nutrient scarcity encourages microcolony formation. The functionality of QS systems also varies with environmental context; QS-deficient strains are unable to swarm or produce organized biofilms in minimal media, yet these capabilities can be restored in nutrient-rich media, possibly due to metabolic adaptations [30].

At the molecular scale, QS controls the expression of genes involved not only in biofilm formation but also in the production of various virulence factors, including proteases, lipases, hemolysins, and a red pigment, prodigiosin [50,53]. QS also regulates bacterial motility patterns such as swarming and swimming, which play essential roles in colonizing the host and advancing the infection [54,55].

Biofilm formation by *S. marcescens* carries important clinical consequences, particularly in cases of IE among PWID. The reasons for the elevated incidence of *S. marcescens* IE in this group remain unclear but may involve factors such as the use of non-sterile tap water during drug preparation, exposure to high bacterial loads during injection, and prior valve damage. Notably, most patients had no history of previous IE episodes, suggesting that contaminated injection equipment, rather than existing valvular abnormalities, plays a central role in infection acquisition [33,56]. This hypothesis is reinforced by harm-reduction research demonstrating that 59% of the 160 used syringes examined contained viable bacteria, including *S. marcescens*, highlighting a likely pathway of transmission and emphasizing the importance of focused public health interventions [57].

Hospital outbreaks of *S. marcescens*, often involving clonal strains, have been reported, particularly in neonatal intensive care units. Strains with enhanced biofilm-forming capabilities contribute to prolonged colonization and infections such as ventilator-associated pneumonia [38].

Closely related species like *S. liquefaciens* also form biofilms, but in this case, environmental factors such as temperature and nutrient levels seem more influential than QS [58].

The formation of biofilms in *S. marcescens* arises from the intricate interaction between QS mechanisms, environmental influences, and genetic determinants. This multifaceted regulatory network enables the bacterium to persist in clinical and community environments, thereby playing a growing role in challenging infections like IE.

### 3.4. Klebsiella pneumoniae

*K. pneumoniae* represents an important clinical pathogen, notable for its capacity to form biofilms and its increasing multidrug resistance, primarily due to enzymes such as extended-spectrum β-lactamases (ESBLs) and carbapenemases.

Type III fimbriae serve as crucial surface appendages, facilitating the initial attachment of *K. pneumoniae* cells and playing a vital role in the development of mature biofilm communities [59]. Additional virulence determinants, including capsular polysaccharides, type I pili/fimbriae, and several adhesin proteins, enhance bacterial adherence to living tissues as well as inanimate surfaces, thereby promoting colonization and disease progression [60].

The biofilm extracellular matrix acts as a shield, hindering antibiotic penetration and chemically inactivating antimicrobial agents, which aids bacterial persistence [61]. Research demonstrates that *K. pneumoniae* cells residing in biofilms exhibit resistance to antibiotics such as ampicillin, despite planktonic cells showing susceptibility under laboratory conditions, a characteristic referred to as biofilm recalcitrance [62]. QS in *K. pneumoniae* coordinates biofilm formation by regulating genes for surface structures and exopolysaccharides, with AI-2 molecules contributing to early microcolony development and the LuxR-type regulator SdiA modulating fimbrial expression, thereby influencing both the architecture and the persistence of biofilms in intra- and interspecies communities [61].

Finally, toxins in *K. pneumoniae* contribute to biofilm-associated pathogenicity by damaging host cells, modulating immune responses, and promoting persistence, with key virulence factors such as hemolysin and cytotoxins enhancing bacterial survival and dissemination during infection [63].

The growing clinical challenge of *K. pneumoniae* infections stems from its capacity to form resilient biofilms combined with the production of resistance enzymes like ESBLs and carbapenemases, significantly narrowing the available therapeutic strategies. Epidemiological studies reveal that around 80% of clinical *K. pneumoniae* strains can form biofilms, which support their sustained presence on medical devices such as catheters and ventilators. This characteristic plays a significant role in infections like catheter-associated urinary tract infections, ventilator-associated pneumonia, and the colonization of prosthetic implants [64].

In summary, the interplay of surface adhesins, protective biofilm architecture, toxin production, and multidrug resistance mechanisms highlight *K. pneumoniae* as a high-risk nosocomial pathogen. Its biofilm-forming ability not only facilitates chronic infection and immune evasion but also complicates therapy, underscoring the need for effective infection control and innovative antimicrobial strategies.

### 3.5. Escherichia coli

The rarity of *E. coli* IE is largely due to its poor capacity for endocardial adhesion and the efficient immune response, particularly antibody-mediated clearance, in healthy hosts [13,65,66]. Most *E. coli* strains are rapidly cleared from the bloodstream by innate immune mechanisms. Nevertheless, certain serum-resistant variants can evade these defenses and remain in circulation. Even so, their limited ability to adhere to cardiac endothelium continues to account for the infrequency of *E. coli*-related IE [27,67].

Interestingly, several clinical observations link *E. coli* IE to preceding or concomitant urinary tract infections (UTIs), implying a potential role for uropathogenic strains in cardiac involvement. In one review, 17 out of 33 reported cases (52%) had a history of UTIs, supporting the involvement of extraintestinal pathogenic *E. coli* (ExPEC) in the pathogenesis [27,65]. Among ExPEC strains, those belonging to phylogenetic group B2 are notable for expressing multiple virulence mechanisms, including adherence proteins, siderophore systems, immune escape strategies, and various toxins. These traits enable them to survive outside the intestinal tract and potentially invade the bloodstream, facilitating the colonization of native heart valves [68,69].

Late diagnosis of *E. coli* IE is frequently associated with worse clinical outcomes. Research indicates that, in 90% of non-HACEK Gram-negative IE cases, symptoms persisted for more than a month prior to identification. This diagnostic delay, often caused by vague clinical signs and low suspicion, may facilitate infection of even structurally intact heart valves by *E. coli* [65,70].

The ability to produce biofilms plays a key role in urinary tract infections, as strains exhibiting enhanced biofilm formation tend to achieve greater colonization and persistence [71]. Both genetic determinants and environmental factors contribute to the robustness and stability of these biofilms [72]. Notably, multidrug-resistant *E. coli* isolates frequently display elevated biofilm production, which may represent an adaptive mechanism to withstand environmental challenges [73,74].

Research indicates that as many as 78% of uropathogenic *E. coli* isolates combine resistance to serum-mediated killing with increased biofilm-forming abilities. Additional virulence traits, including type 1 pili-driven hemagglutination, gelatinase production, heightened surface hydrophobicity, and motility, contribute to their persistence in hostile environments, and promote the initial attachment during biofilm formation [73,75].

In summary, while *E. coli* IE remains rare, serum-resistant, biofilm-forming uropathogenic strains with multiple virulence factors may overcome host defenses, enabling persistence, bloodstream invasion, and the occasional colonization of cardiac valves.

**Table 2:** Selected pathogenetic features of NHGN.

Pathogen	Entry and Source	Adhesion and Colonization	Biofilm Features	Other Pathogenesis Notes
***Pseudomonas aeruginosa***	Healthcare-associated infections Device-related infections	Type IV pili, flagella, adhesins Alginate enhances binding	Complex (flat or tower/mushroom) Alginate and rhamnolipids matrix QS-regulated (las, rhl systems) Architecture influenced by environmental factors	Toxins (exotoxin A, elastases, alkaline protease, hemolytic phospholipase C) Dispersal triggered by environment
***Serratia marcescens***	Injection drug use Healthcare-associated infections Device-related infections	Type I fimbriae (fimA, fimC) Extracellular polymeric substances aids device adhesion	Porous, filamentous QS-regulated Serrawettin-enhanced development Nutrient-dependent	QS controls proteases, lipases, hemolysins, prodigiosin and motility Injection equipment colonization in IDU
***Klebsiella pneumoniae***	Healthcare-associated infections	Type I/III fimbriae Capsular polysaccharides Adhesins	High prevalence of biofilm producers QS for intra- and interspecies communication	Multidrug resistance enzymes (ESBL, carbapenemases) Toxin production (hemolysins, cytotoxins) Antibiotic recalcitrance
***Escherichia coli***	Urinary tract infections	Poor cardiac endothelium adhesion Type I fimbriae ExPEC adherence proteins	High biofilm production in uropathogenic strains Increased in serum-resistant and MDR	ExPEC (especially B2 strains): siderophores, toxins Immune evasion

QS—quorum sensing; ESBL—Extended-Spectrum Beta-Lactamase; ExPEC—Extraintestinal Pathogenic *Escherichia coli*; MDR—multidrug resistant.

## 4. Diagnosis

### 4.1. Evolution of Diagnostic Criteria for Infective Endocarditis

IE is typically diagnosed according to the Duke Criteria, which were originally published in 1994 [76] and were modified in 2000 [77]. However, since the publication of these criteria, substantial advances have been made in our microbiological understanding, diagnostic approaches, epidemiology, and the management of IE. Consequently, in 2015, the European Society of Cardiology (ESC) proposed revisions to the modified Duke Criteria [78]. Building upon these revisions, further updates were introduced in 2023, when the International Society for Cardiovascular Infectious Diseases (ISCVID) published a new version of the diagnostic criteria for IE [20], and the final version of the ESC Guidelines for the management of IE [79] introduced several important updates, incorporating recent genetic, molecular, and tissue staining techniques for the detection of etiologic microorganisms as well as updates to the clinical criteria, particularly in imaging and microbiological domains.

### 4.2. Advances in Microbiological and Molecular Diagnostics

#### 4.2.1. Expanded List of Typical Pathogens

With regard to microbiological criteria, ISCVID suggested the inclusion of additional organisms as “typical” pathogens in the setting of intracardiac prosthetic material, such as coagulase negative staphylococci, *Corynebacterium striatum* and *Corynebacterium jeikeium*, *S. marcescens*, *P. aeruginosa*, *Cutibacterium acnes*, nontuberculous mycobacteria (particularly *Mycobacterium chimaera*), and *Candida* spp. [20]. Notably, this marked the first time that Gram-negative bacteria were formally recognized as typical causative agents of IE. Conversely, the 2023 ESC Guidelines [79] do not classify non-HACEK Gram-negative bacteria as “typical” causative agents of IE. However, they are acknowledged as potential etiological agents of IE, particularly in cases involving cardiovascular implantable electronic device (CIED)-related IE, as well as in patients admitted to intensive care units, people who inject drugs, and immunocompromised individuals.

#### 4.2.2. Diagnostic Criteria and Imaging Updates

In the 2023 ESC guidelines, the diagnostic approach was also refined, with particular emphasis on imaging findings [79]. Anatomical lesions and increased uptake observed on [18F]-fluorodeoxyglucose positron emission tomography/computed tomography (18F-FDG PET/CT) are now considered major criteria. In addition, abnormal prosthetic or periprosthetic uptake—specifically intense focal or heterogeneous uptake—detected via 18F-FDG PET/CT or white blood cell (WBC) SPECT/CT is regarded as a major criterion for PVE, regardless of the interval from surgery.

Similarly, the ISCVID criteria [20] included 18F-FDG PET/CT as a major imaging criterion. Interestingly, in patients with cardiovascular devices, uptake detected withing 3 months of surgical intervention was considered as a minor imaging criterion.

Finally, in both 2023 ESC guidelines and ISCVID criteria [20,79], abnormal findings at cardiac computed tomography (CT) were also included as major imaging criteria.

#### 4.2.3. New Molecular Techniques

The 2023 Duke-ISCVID Criteria [20] emphasize additional microbiological tests that may achieve major criterion status, particularly in the context of blood-culture-negative endocarditis (BCNIE). Specifically, the ISCVID update highlights two more recent molecular approaches as valuable tools for identifying the etiology of BCNIE: amplicon sequencing (e.g., 16S, 18S rRNA, or internal transcribed spacer regions) and hypothesis-free metagenomic (‘shotgun’) sequencing. Accordingly, the ISCVID Working Group recommends that a positive result obtained through amplicon or metagenomic sequencing from cardiac tissue, cardiac prosthesis, or arterial embolus should be considered a major criterion. Further, positive findings from a sterile body site other than cardiac tissue, cardiac prosthesis, or arterial embolus should be considered as a microbiological minor criterion, as a single isolated detection of a skin bacterium on a valve or wire, in the absence of corroborating clinical or microbiological evidence.

In the 2023 ESC Guidelines [79], the role of molecular diagnostics is also acknowledged, although in broader and less prescriptive terms. Specifically, the use of 16S/18S rRNA PCR sequencing on cardiac tissue or embolic material is recognized as a valuable tool to enhance diagnostic accuracy in cases of BCNIE; however, it continues to be classified as a minor criterion.

### 4.3. Comparative Performance of Diagnostic Criteria: Sensitivity and Specificity

With the updates of the Modified Duke Criteria, several studies have compared the 2015 ESC guidelines [79] to the 2023 Duke-ISCVID Criteria [20], with the aim of assessing the impact of the updated criteria on the diagnostic classification of IE.

In 2023, the DERIVE registry conducted a retrospective analysis of patients with a diagnosis of IE recorded between November 2019 and December 2023 [80]. The findings from this study indicated that the 2023 Duke-ISCVID Criteria demonstrated improved sensitivity compared to the 2015 ESC Modified Duke Criteria, leading to a reclassification of several cases previously categorized as “possible IE” to “definite IE”. The primary factor contributing to this increased diagnostic sensitivity was the incorporation of molecular diagnostic tools, particularly PCR testing performed on explanted valvular tissue. Notably, in this subset of reclassified cases, no non-HACEK Gram-negative pathogens were identified, underscoring that the diagnostic gain was primarily attributable to enhanced detection of traditionally recognized IE organisms through advanced microbiological techniques rather than to a broader inclusion of non-HACEK Gram-negative species.

In line with these findings, a study conducted on patients enrolled in the Observatoire EI registry and treated for IE between January 2017 and October 2022 [81] demonstrated that the updated pathological criteria significantly contributed to the increased sensitivity of the 2023 Duke-ISCVID Criteria compared to both the 2000 Modified Duke and the 2015 ESC criteria. In particular, the higher number of cases meeting major pathological criteria under the 2023 classification was primarily due to improved pathogen detection, with 16.4% of pathogens detected in appropriate samples using advanced molecular techniques, including PCR, amplicon, or metagenomic sequencing, and in situ hybridization. Despite the observed improvement in sensitivity, the study also reported a concomitant reduction in specificity when applying the 2023 Duke-ISCVID Criteria.

Additional evidence supporting the improved sensitivity of the 2023 Duke-ISCVID Criteria comes from a study conducted at the Lausanne University Hospital and the University Hospital Zurich, which analyzed cohorts of patients with suspected IE between 2014–2017 and 2018–2022 [82]. In this evaluation, the updated Duke-ISCVID 2023 Criteria contributed to an increase in sensitivity, with more cases being classified as “definite IE”, mainly thanks to the expanded the role of 18F-FDG PET/CT and the updated microbiological definitions. However, as mentioned in the previous study [81], this modification was not without drawbacks: while it led to a modest gain in sensitivity, it also resulted in a reduction in specificity, raising concerns about potential overdiagnosis. The authors of the study emphasized that this could contribute to an increase in unnecessary diagnostic procedures, including transthoracic or transesophageal echocardiography.

A study that underscores the clinical relevance of the updates to both the major microbiological and imaging criteria is the one conducted in Amsterdam between 2016 and 2021 [83]. In this analysis, an international expert panel independently reviewed case summaries and adjudicated each case as either “IE” or “not-IE”. Using the panel’s diagnosis as the reference standard, the 2023 Duke-ISCVID Criteria demonstrated similar sensitivity, but higher specificity compared to the 2023 ESC Criteria. When compared to the 2015 ESC Criteria, the 2023 Duke-ISCVID Criteria showed greater sensitivity, while maintaining comparable specificity. Notably, the increased sensitivity observed in this study was primarily attributed to the inclusion of updated microbiological and imaging criteria. However, the study did not detail the specific pathogens involved in the reclassified cases, limiting the ability to determine how many of these were attributable to Gram-negative bacteria.

Further evidence supporting the impact of the updated diagnostic criteria was provided by a retrospective study conducted in Sweden [84], which analyzed 4050 episodes of bacteremia to assess the effects of implementing the revised list of microorganisms now classified as “typical’” causes of IE in the 2023 Duke-ISCVID Criteria. In this analysis, reclassification from “possible” to “definite IE” and from “rejected” to “possible IE” was largely driven by a limited number of newly designated pathogens available for inclusion (specifically *Streptococcus dysgalactiae*, *S. lugdunensis*, and *Gemella* species) potentially underestimating the broader diagnostic impact of the updated classification.

Taken together, while the incorporation of advanced microbiological techniques has clearly enhanced the sensitivity of available criteria, several studies have also demonstrated a reduction in specificity, raising concerns about possible overdiagnosis and misclassification. This underscores the importance of careful interpretation of microbiological results, ideally within a multidisciplinary endocarditis team, to ensure that these tools are used judiciously. In particular, their greatest clinical value may lie in patients with prior antibiotic exposure, culture-negative endocarditis, or unclear etiology despite standard microbiological investigations, where they can provide decisive diagnostic clarification without disproportionately increasing the risk of false positives.

### 4.4. Focus on NHGNIE in Recent Studies

Two recent studies [8,85] have evaluated the performance of various versions of the Duke clinical criteria (including the 2015 ESC Criteria [78], the 2023 ESC Criteria [79], and the 2023 Duke-ISCVID Criteria [20]) for the diagnosis of IE in patients with bacteremia or candidemia due to newly recognized typical pathogens, including non-HACEK Gram-negative bacteria.

One of these studies, conducted at Lausanne University Hospital in Switzerland between January 2014 and March 2023 [85], classified microorganisms into two distinct categories: Group A included pathogens considered typical regardless of the presence of intracardiac material (e.g., *S. lugdunensis*, *Granulicatella* spp., *Abiotrophia* spp., and *Gemella* spp.); Group B encompassed microorganisms considered typical only when intracardiac material was present, such as coagulase-negative staphylococci other than *S. lugdunensis*, *Cutibacterium acnes*, *Corynebacterium striatum*, *C. jeikeium*, *P. aeruginosa*, *S. marcescens*, non-tuberculous mycobacteria, and *Candida* spp. The episodes were retrospectively assessed and reclassified according to the 2015 ESC criteria, the 2023 ESC criteria, and the 2023 Duke-ISCVID Criteria. Among the 939 episodes analyzed, the most frequently isolated newly typical pathogens were *Candida* spp. (128 episodes; 28%), followed by *S. epidermidis* (125; 27%) and *P. aeruginosa* (94; 20%). The application of the 2023 Duke-ISCVID Criteria resulted in a higher rate of fulfillment of the major microbiological criterion compared to both ESC versions, thereby demonstrating improved sensitivity in identifying IE cases. Notably, this finding contrasts with results from the Amsterdam cohort study [83], in which the sensitivity of the 2023 Duke-ISCVID and 2023 ESC criteria appeared comparable. The authors of the Swiss study [85] suggest that this discrepancy may be attributed to the different patient populations evaluated: while the Amsterdam study focused on individuals with suspected IE, the Lausanne study investigated pathogens that, although newly classified as typical, still account for only a minority of IE cases.

Finally, a study based on data from the Swedish Registry of Infective Endocarditis between 2008 to 2023 [8], assessed the impact of the inclusion of *P. aeruginosa* and *S. marcescens* as “typical” pathogens in the 2023 Duke-ISCVID Criteria. The analysis did not demonstrate an improvement in diagnostic sensitivity resulting from this update. However, the authors noted that only a very small number of IE cases in the Swedish cohort were attributable to these pathogens, which may limit the ability to detect a meaningful effect and potentially underestimates the clinical relevance of this revision in other settings. Moreover, the authors emphasize that three episodes of “possible IE” caused by other non-HACEK Gram-negative organisms (*E. coli* and *Neisseria* spp.) would have been reclassified as “definite IE” if the 2023 Duke-ISCVID Criteria had included all non-HACEK Gram-negative bacteria as “typical” pathogens in the context of intracardiac prosthetic material.

Overall, although the updated diagnostic frameworks have been associated with improved sensitivity across different cohorts, the available evidence specifically addressing non-HACEK Gram-negative pathogens remains limited. In most comparative studies, either pathogen-specific data were not reported or the number of IE cases caused by these organisms was extremely small, limiting the ability to meaningfully assess the impact of their inclusion as “typical” in the 2023 Duke-ISCVID Criteria. These limitations underscore the need for larger, multicenter studies focusing on these subgroups of pathogens to better define their epidemiological burden, diagnostic implications, and potential influence on patient management.

## 5. Clinical Features and Outcomes

NHGNIE displays a distinct and variable clinical profile, shaped significantly by underlying epidemiologic factors, including patient demographics, healthcare exposure, injection drug use, and regional antimicrobial resistance patterns. The disease course is often subacute, yet complications are frequent, and outcomes vary considerably depending on region- and population-specific factors. The clinical characteristics and outcomes of patients with NHGNIE from selected cohorts are described in Table 3.

### 5.1. Fever

Fever, although a hallmark of IE, is less consistently observed in NHGNIE than in other etiologies. In classical IE cohorts [86,87], fever is described in 78–95% of patients. By contrast, NHGNIE cases show greater variability, with reported frequencies ranging from 58 to 100% [1,2,5,6,9,10,11,13,16]. The largest NHGNIE cohort to date, dominated by PWID (52%), documented fever in only 61% of patients [2]. Notably, fever was less frequently reported in infections caused by *Serratia* spp. and *P. aeruginosa*, which were more common among PWID, than in cases due to *Enterobacterales*. Conversely, in elderly populations with high comorbidity burdens, such as the ICE-PCS international cohort where PWID comprised only 4%, fever remained more prevalent, reaching 92% of cases [1].

### 5.2. Valvular Vegetations and Intra-Cardiac Abscesses

The detection of valvular vegetations in patients with NHGNIE is variable, ranging from 69 to 92% across studies [1,3,10,11,14]. Importantly, NHGNIE is frequently associated with large vegetations, with median sizes exceeding 10 mm reported in several cohorts [2,3,5,13,14]. Intracardiac abscesses were documented in 3% to 42% of NHGNIE cases [1,5,6,7,9,10,11,14]. In the ICE-PCS cohort, patients with NHGNIE had a significantly higher rate of intracardiac abscesses than those with IE of other etiologies [1]. Notably, in a study from Serbia examining the impact of etiology on echocardiographic features, NHGNIE (the majority of which were due to *P. aeruginosa*) cases were shown to be associated with large (>15 mm) vegetations and perivalvular abscess or pseudoaneurysm [88].

### 5.3. Heart Failure

Heart failure was present in 8% to 47% of NHGNIE cases across studies [1,2,5,6,9,10,11,12,13,14]. Interestingly, in some studies [11,14], heart failure was shown to be less frequent in patients with NHGNIE, possibly reflecting differences in patients population and microbial virulence. Interestingly, among patients with NHGNIE, *P. aeruginosa* etiology was shown to be more frequently associated with heart failure [2].

### 5.4. Embolic Events

Embolic complications are a central feature of NHGNIE, occurring in 10–66% of cases [1,2,3,4,5,6,7,8,9,10,11,12,13,14,16]. Interestingly, rates of embolization were especially high in PWID-based cohorts from the U.S. [2,4], where septic emboli occurred in up to 66% of patients. By contrast, embolic events were less frequent in cohorts comprised primarily of elderly patients with comorbidities, suggesting possible underdiagnosis or pathogen-related differences in embolic potential. Notably, a study from Sweden found that embolization rates in NHGNIE were comparable to *S. aureus* IE (32%), but higher than those seen in streptococcal and enterococcal IE. Conversely, a French study [9] reported fewer embolic complications in NHGNIE (31%) compared to other etiologies.

### 5.5. Septic Shock

Few studies reported on the proportion of patients with NHGNIE with septic shock, with rates ranging from 3% to 40% [4,7,9,11,12,14]. A multicenter study from Spain [11] described a significantly higher frequency of septic shock in NHGNIE patients (21%) compared to other IE etiologies. Additionally, in a study including 4864 patient with IE, etiology due to Gram-negative rods was identified as an independent predictive factor of septic shock [89].

### 5.6. Relapses and Recurrences

Relapse and recurrence rates in patients with NHGNIE range between 1% and 15% [2,4,10,11]. In the largest study available to date [2], microbiological failure occurred in 10% of patients, with 15% recurrence among survivors. Interestingly, 83% of microbiological failures were associated with development of resistance and were more common in patients with *P. aeruginosa* (23%). Nevertheless, the distinction between relapse and recurrence is not always clearly defined, and patients with specific risk factors (e.g., PWID) may be more prone to recurrences than relapses. When comparing Gram-negatives with other etiologies, a recent study evaluating the proportion of persistent bacteremia among patients with non-staphylococcal IE showed that only 7 out of 159 patients had persistent bacteremia, of whom 5 cases were due to *Enterococcus faecalis* and 2 cases were due to Gram-negative bacteria: *P. aeruginosa* and *Enterobacter cloacae* [90].

### 5.7. Mortality

Mortality in NHGNIE shows substantial geographic and demographic variation, with in-hospital mortality ranging from 0% to 41%, and 1-year mortality reaching up to 44% [1,2,3,4,5,6,7,8,9,10,11,12,13,14,15,16]. Cohorts with younger, PWID-dominant populations generally reported lower in-hospital mortality despite high complication rates. In contrast, studies involving older, comorbid patients tended to show more frequent fatal outcomes. The impact of MDR pathogens on prognosis may be especially notable. A recent review focusing on this MDR Gram-negative IE reported a mortality of 38.5% [25], and data from Italy identified MDR etiology as a significant independent predictor of mortality [14].

### 5.8. Pathogen-Specific Profiles in NHGNIE

The clinical behavior, risk factors, and outcomes of NHGNIE differ substantially by pathogen. Understanding species-specific characteristics is critical for optimizing diagnostic suspicion, therapeutic strategies, and prognostic assessment. This section highlights current evidence on the most commonly implicated organisms: *Pseudomonas* spp., *Serratia* spp., and other *Enterobacterales*.

#### 5.8.1. *Pseudomonas* spp.

*Pseudomonas* spp. is a major cause of NHGNIE and is associated with both healthcare-associated infections and IE in PWID, and the clinical significance of *P. aeruginosa* in device-related infections has been increasingly recognized. A recent U.S. study highlighted a high risk of IE among patients with prosthetic valves [19], prompting the inclusion of this pathogen in the list of typical microorganisms in the updated Duke-ISCVID Criteria [20].

The largest study regarding *P. aeruginosa* IE to date included 48 patients diagnosed between 2000 and 2024 in a U.S. hospital setting [91]. PWID (29%) and transplant recipients (13%) were frequent; most patients had native valve endocarditis (NVE, 67%) with large vegetations (52% over 10 mm); septic embolization was common (56%). Interestingly, 13% had MDR isolates at the diagnosis, with an additional 20% developing resistance during therapy. The 90-day mortality rate was 29%.

In a Australian multicenter study spanning 20 years [92], 15 cases were reported, mostly healthcare-associated (60%) but also frequent in PWID (40%). Prosthetic valves were often involved (27%), and surgery was common (47%). In-hospital mortality was 13%, increasing to 27% at 1 year.

A review [93] including 218 cases of *Pseudomonas* spp. IE reported *P. aeruginosa* as the dominant pathogen (92%). PWID was the most common risk factor (38%), with end-stage renal disease or hemodialysis also being frequent (13%). Embolic events were common (32%, 17% stroke). MDR strains were identified in 20% of patients. Overall mortality reached 26%.

In the largest NHGNIE cohort [2], *P. aeruginosa* was the second most frequent pathogen (21%). These patients had higher rates of PVE (31%), persistent bacteremia (23%, median duration of 10 days), and microbiological failure (23%) compared to other Gram-negative organisms. In another U.S. study, *Pseudomonas* spp. was shown to be more common in PWID (11% of cases) [94].

Resistance remains a clinical concern. A recent U.S. cohort [4] reported MDR rates of 10% among *Pseudomonas* IE cases. Furthermore, *Pseudomonas* etiology was independently associated with higher risk of mortality and hospital readmission, emphasizing its role as a high-risk pathogen.

#### 5.8.2. *Serratia* spp.

*S. marcescens*, the most common *Serratia* species associated with IE, has emerged as an important cause of NHGNIE, particularly in younger populations and among PWID. As with *Pseudomonas*, *Serratia* spp. have been added to the “typical pathogen” list in the Duke-ISCVID Criteria [20], based on prospective data demonstrating similar prosthetic valve infection risk to *S. aureus* in bacteremic patients [19].

A recent study conducted in the U.S. [95] described the largest cohort of *Serratia*-associated IE, comprising 159 cases between 2015 and 2021. The majority of cases were among people under 40 years of age (67%), and 75% were PWID. Most patients had NVE (69%), and the in-hospital mortality or discharge to hospice rate was 21%.

Another multicenter study from the U.S. [96] identified 75 cases of *Serratia* IE (mostly *S. marcescens*), with 85% PWID. PVE was observed in 21% of cases. Large vegetations (over 10 mm in 71%) and septic embolization (76%) were frequent. Clinical failure (24%) and relapse (12%) were frequent.

In the largest NHGNIE cohort [2], *Serratia* spp. was the most common pathogen (43%), mainly seen in young patients with few comorbidities and high PWID prevalence (85%). Large vegetations were common (>10 mm in 77%), but fever (49%) and heart failure (19%) were less frequent.

Further, a notable study on IE in pregnancy [97] identified *Serratia* spp. as the second most frequent pathogen (13%) after *S. aureus*. However, all patients were PWID.

From an epidemiological point of view, the strong association between *Serratia* spp. and PWID may be linked to the use of non-sterile water (e.g., colonized tap water) for injection preparation, though this hypothesis remains speculative [98].

#### 5.8.3. Other *Enterobacterales*

Other *Enterobacterales*, including *E. coli* and *Klebsiella* spp., are often encountered in older patients with significant comorbidities.

In the largest available NHGNIE cohort [2], compared to patients with IE due to other Gram-negative bacilli, patients with *Enterobacterales* were older, with a higher comorbidity burden, and were more likely to have liver cirrhosis.

A multicenter Spanish study [11] compared *Enterobacterales* to non-fermenting Gram-negative bacilli, showing that patients with *Enterobacterales* IE had more community-acquired infections (56%), fewer catheter-related infections (17%), and a predominance of genitourinary sources (33%). Right-side valve involvement was rare (6%), while septic shock occurred more often (28%).

Finally, in a Brazilian study [5], mortality among patients with *Enterobacterales* IE was significantly lower than among those with non-fermenting Gram-negative (35% vs. 75%), emphasizing differences in virulence and host susceptibility.

## 6. Management

### 6.1. Antimicrobial Treatment

Defining standard antimicrobial therapy for NHGNIE is a difficult task due to the rarity of these infections, their heterogeneity, and the potential for multidrug resistance. In this scenario, consultation with experienced infectious diseases specialists is strongly advised [99].

According to current guidelines [79,99] (shown in Table 4), early surgical therapy and combination therapy consisting of a beta-lactam (such as penicillin, cephalosporins, or carbapenems) and either an aminoglycoside or fluoroquinolone for 6 weeks are often considered acceptable strategies. However, it must be emphasized that these are primarily based on expert opinions rather than high-quality evidence [100]. The biological rationale for combining beta-lactams with aminoglycosides or fluoroquinolones in NHGNIE is based on their complementary mechanisms of action: beta-lactams disrupt the cell wall, aminoglycosides inhibit protein translation, and fluoroquinolones interfere with DNA replication. Early in vitro and experimental studies suggested frequent synergy with these regimens, particularly against *P. aeruginosa*, leading to the concept that such combinations could enhance bactericidal activity and reduce resistance emergence [101,102,103,104,105,106]. More recent systematic studies, however, have shown that true synergy is uncommon and most combinations are indifferent, with occasional antagonism [107,108,109]. For *Enterobacterales*, this limited or absent synergy is consistent across both aminoglycoside and quinolone partners [107,108,110], whereas for *P. aeruginosa*, synergy is more often observed, particularly with beta-lactam–aminoglycoside combinations, though still variable and method-dependent [110,111,112]. Notably, some studies have also highlighted the potential of these regimens to inhibit biofilm formation in *P. aeruginosa* [113,114]. Overall, the biological rationale remains grounded in mechanistic complementarity, occasionally in in vitro synergy, and in its potential effects on biofilms.

A summary of recent reports evaluating the impact of combination therapy (mainly beta-lactams in combination with aminoglycosides of fluoroquinolones) is shown in Table 5.

In most studies, no difference in outcome was reported when comparing patients treated with combination therapy compared to those receiving monotherapy. In the largest available study, which included 123 NHGNIE cases, 43% were treated with combination therapy, without a significant association with better outcomes on multivariate analysis [2]. A recent single-center retrospective study including 60 with NHGNIE found no significant difference in 60 day-mortality, readmission, or microbiological failure between patients who received monotherapy and those who received combination therapy [3]. Notably, adverse events occurred more frequently with combination therapy (19% vs. 0%, *p*-value 0.012). Finally, the ICE-PCS study, which included 49 patients (63% treated with combination therapy), no mortality difference was observed when comparing combination therapy with monotherapy [1]. Taken together, these data suggest that routine use of combination therapy may not confer a survival advantage and may increase toxicity.

However, other studies demonstrated an association between combination therapy and outcomes. In a large study from Spain [11], including 104 patients with NHGNIE, 79% received combination therapy. After adjusting for potential confounders, in-hospital mortality appeared to be lower among patients who received combination therapy with beta-lactams and fluoroquinolones (Adjusted Odds Ratio (aOR)—0.29; Confidence Interval (CI)—0.09–0.96). Conversely, a U.S. retrospective cohort of 43 patients (76% treated with combination therapy) showed that fluoroquinolone-based combination regimens were associated with higher rates of mortality and readmission at 90 days (aOR 6.7, CI 1.4–30.4) and higher readmission rates at 12 months (aOR 33.9, CI 2.7–429.9) [4]. Thus, the available evidence on combination therapy is conflicting, with geographic and methodological differences likely contributing to heterogeneity in results.

Interestingly, an Italian multicenter study including 58 patient with NHGNIE (86% treated with combination therapy) found at univariate analysis that the use of non-carbapenem beta-lactams (penicillin or cephalosporins) as backbone agents was associated with improved survival [14], highlighting the potential relevance of the beta-lactam backbone, although confirmatory studies are lacking.

Some studies further explored the role of combination therapy in specific pathogens. In the largest available cohort from the U.S. [2], a potential benefit of combination therapy was noted for *Serratia* and *Enterobacterales*; meanwhile, for *P. aeruginosa*, mortality was higher among those treated with combination regimens. In a cohort of 48 patients with *P. aeruginosa* endocarditis, combination therapy (used in 69% of patients) conferred no survival benefit and was linked to higher rates of adverse events leading to drug discontinuation (21% vs. 0%, *p*-value 0.08) [91]. A smaller retrospective series of 15 cases reported combination therapy in 53% of cases, with one-year mortality numerically higher in the monotherapy group (75% vs. 36%, *p*-value 0.282) [92]. A systematic review of 218 cases of *P. aeruginosa* endocarditis found lower mortality with quinolone-based regimens (10.3% vs. 28.6%, *p*-value 0.04), but no difference between monotherapy and combination therapy [93]. Altogether, evidence in *P. aeruginosa* endocarditis points to higher toxicity with combination regimens and inconsistent survival benefits, underscoring the need for individualized treatment decisions.

In the largest cohort of *Serratia* IE, including 159 patients, 43% received combination therapy, which was associated with reduced inpatient mortality (aOR 0.15, CI 0.03–0.74) [95]. In another U.S. cohort of 75 patients, 48% received combination therapy, which was associated with lower microbiological failure (0% vs. 15%, *p*-value 0.026), reduced 90-day mortality (11% vs. 31%, *p*-value 0.049), and lower rates of clinical failure (aOR 0.17, CI 0.03–0.86). However, adverse events leading to drug discontinuation were numerically more common (36% vs. 8%, *p*-value 0.058). Notably, patients treated with ampC-inducing regimens (such as ceftriaxone, piperacillin/tazobactam or ceftazidime) had outcomes comparable to those on ampC-non-inducing agents [96]. These results suggest a possible benefit of combination therapy in *Serratia* IE, although the risk of adverse events must be carefully weighed.

Fewer data are available regarding optimal treatment of other NHGN pathogens. As many Gram-negative species harbor diverse resistance mechanisms, in vitro susceptibility testing is essential to guide therapy [99]. In *Enterobacterales* endocarditis, therapy should be guided by susceptibility testing, and early surgery considered in difficult-to-treat infections with limited treatment options, since the likelihood of treatment failure is high [115]. The increasing prevalence of ESBL-producing pathogens underscores this need. Successful therapy with carbapenem monotherapy has been reported for patients affected by ESBL-producing *E. coli* endocarditis [65], though some Authors recommend higher-dose regimens [56].

Data on endocarditis caused by MDR pathogens remain limited. These infections usually occur in nosocomial settings, often in fragile patients with significant comorbidities. Aggressive medical treatment combined with prompt surgery is recommended, as prognosis is generally poor, especially when compared to that of patients with IE due to pathogens with a more favorable susceptibility profile [14,25,116].

Rare and atypical NHGNIE pathogens, such as *Salmonella* spp., *Acinetobacter* spp., *Stenotrophomonas maltophilia*, *Pasteurella multocida*, *Capnocytophaga* spp., *Neisseria* spp., and *Achromobacter xylosoxidans* [115,117,118,119,120,121,122,123], are mainly described in small case series or reports, with management relying on pathogen-directed regimens (often beta-lactam-based combinations with aminoglycosides, fluoroquinolones, or trimethoprim–sulfamethoxazole), guided by susceptibility testing. Outcomes vary, but surgical intervention and device removal are frequently required, particularly for resistant or refractory infections.

Currently, there is no consensus on transition to oral therapy for NHGNIE, and such patients were excluded from the largest oral switch study [124]. Notably, Shah et al. reported oral suppressive therapy in 13% of patients with NHGNIE (trimethoprim–sulfamethoxazole or fluoroquinolone) [2]. Older reports describe trimethoprim–sulfamethoxazole as part of oral regimens, mostly for unusual pathogens such as *Coxiella burnetii*, *Burkholderia cepacia*, and *Stenotrophomonas maltophilia* [125].

In summary, management of NHGNIE requires individualized therapy guided by pathogen-specific susceptibility, careful balancing of combination versus monotherapy, and consideration of early surgical intervention in refractory or drug-resistant cases.

### 6.2. Surgical Treatment

According to the ESC guidelines, surgical treatment is indicated in cases of heart failure, high embolic risk, or uncontrolled infections [79]. Infection is considered uncontrolled when bacteremia persists (positive blood cultures for more than one week despite appropriate antimicrobial therapy), when imaging reveals local progression (e.g., increased vegetation size or perivalvular involvement), or when pathogens are unlikely to be eradicated with available agents. This latter group includes fungi, resistant organisms such as methicillin-resistant *S. aureus* (MRSA), vancomycin-resistant *Enterococcus* (VRE)*,* and non-HACEK Gram-negative bacilli [79]. The AHA guidelines provide broadly similar recommendations, but microbiological criteria are more restrictive: surgery is recommended only for fungal infections, resistant Gram-positives such as VRE, and MDR Gram-negative bacilli [99].

Therefore, both ESC and AHA guidelines consider surgery mandatory for Gram-negative prosthetic endocarditis, given that surgery can achieve microbiological eradication more reliably than antimicrobial therapy alone and may mitigate the risk of antimicrobial resistance development [78,126]. However, multiple cohorts investigating the role of surgery in NHGNIE have yielded heterogeneous results.

Studies evaluating the impact of surgery on outcome are described in Table 6.

A Swedish registry of 114 cases of NHGNIE reported that these patients underwent surgery more frequently than those with *S. aureus*, enterococcal, or streptococcal IE. Nevertheless, mortality was higher compared with HACEK, enterococcal, and streptococcal IE. Notably, most NHGNIE cases were associated with multiple comorbidities and healthcare-associated acquisition, which may independently contribute to poorer outcomes [8]. Several studies have evaluated mortality in patients managed surgically compared to those treated conservatively, reporting no significant difference in relevant outcomes [1,4,5,6,7,9,13].

In contrast, evidence consistently shows improved outcomes when surgery is performed according to guideline-based indications, compared with cases where surgery is withheld despite indication. A large U.S. cohort of 123 patients [2] reported that surgery was performed in 40%, with 35 out of 84 patients (42%) not undergoing surgery despite formal indication. Failure to perform surgery despite indication was independently associated with clinical failure (aOR 7.44, CI 2.37–23.4, *p*-value 0.001) and 90-day mortality (aOR 14.6, CI 4.62–45.9, *p*-value < 0.001). Similarly, a Spanish study of 104 patients (surgical indication in 64 [62%], surgery performed in 47 [76%]) corroborated these findings, demonstrating significantly lower mortality when surgery was performed according to guideline-based criteria (27.7% vs. 63.2%, *p*-value 0.007), with multivariate analysis confirming the non-performance of surgery despite indication as a risk factor for mortality (aOR 3.60, CI 1.17–11.05) [11]. An Italian cohort of 58 patients, where 43% underwent surgery, reported no overall difference in mortality between surgically and medically managed patients. However, among those with cardiac complications such as abscess, fistula, dehiscence, valve perforation, or de novo heart failure, mortality was significantly higher with antibiotic therapy alone (60% vs. 18%, *p*-value 0.049) [14].

Pathogen-specific outcomes are equally variable. In a cohort of 159 cases of *Serratia* IE, surgery (performed in 36% of patients) was independently associated with reduced mortality (aOR 0.14, CI 0.03–0.64) [95]. Another *Serratia* IE series found that surgery was indicated in 77% patients and performed in 59% of them, with lack of surgery despite indication associated with increased risk of clinical failure (aOR 3.84, CI 4.5–105) [96].

Conversely, in a series focusing on *P. aeruginosa* IE, surgery was indicated in 63% and performed 57%, but outcomes did not differ significantly between surgical and medical therapy [91]. In contrast, a systematic review reported that surgical intervention in *P. aeruginosa* IE (performed in 57% of cases) was independently associated with lower mortality (aOR 0.41, CI 0.20–0.82) [93].

A key limitation of current guidelines is the lack of a precise definition of MDR in NHGN pathogens. In practice, the conventional definition is applied, classifying microorganisms as MDR when they are resistant to at least one agent in three or more antimicrobial classes [127]. Although IE due to MDR pathogens has been associated with worse outcomes and higher mortality in some studies [14], robust evidence supporting surgery as a universal indication is lacking, particularly when effective antimicrobial options are available. Moreover, recent advances, including the introduction of novel beta-lactam-based agents active against MDR organisms, have substantially improved outcomes in bloodstream infections, often resulting in prognoses comparable to those of infections caused by susceptible strains when optima therapy is used [128]. The scenario changes in cases of pan-resistant organisms, where surgery may represent the only feasible means of infection control [115]. Consequently, the decision to pursue surgical intervention should not be based solely on antimicrobial resistance profiles, but rather carefully individualized, taking into account the patient’s clinical evolution and the availability of effective antimicrobial therapy.

In summary, while current guidelines endorse surgical intervention in NHGNIE underdefined conditions, real-world data demonstrate heterogeneous outcomes. Optimal management requires individualized decision making that integrates microbial susceptibility, patient comorbidities, and clinical progression within the context of current guideline recommendations.

## 7. Future Directions and Unmet Needs

### 7.1. Improved Epidemiological Understanding

While still representing a minority of all IE cases, NHGNIE incidence is increasing, potentially due to the growing population at risk, such as patients with indwelling cardiovascular devices, elderly hospitalized patients with comorbidities, and PWID. Nevertheless, globally consistent data regarding NHGNIE remain scarce, and a comprehensive understanding of its epidemiology is lacking. Notably, there are substantial geographical variations among different case series, potentially related to differences in patient characteristics, but possibly also due to unexplored confounding factors. In this regard, there is a pressing need for large, multicenter, prospective registries to capture incidence, risk factors, and outcomes of NHGNIE. On the one hand, large registries dedicated to IE as a whole, such as ICE-PCS or EURO-ENDO [86,87], represent valuable resources for describing IE epidemiology; on the other hand, given their comprehensive nature, they fail to capture specific features unique to NHGNIE. Moreover, while several studies have explored the factors associated with IE during specific bloodstream infections (such as *S. aureus*, *Enterococcus faecalis*, *Streptococcus* spp. [129]), only a few have evaluated the proportion of patients with IE among those who develop bacteremia due to Gram-negative bacteria. Such data would be useful to better stratify patients and help identify those at risk who require further evaluations to exclude IE.

### 7.2. Diagnostic Advances

While the use of novel or recently introduced microbiological tools, such as metagenomics, is already part of the endocarditis diagnostic process and is well-recognized [20,79], for NHGNIE, the greatest barrier is represented by the timely evaluation of patients for potential endocarditis in the context Gram-negative bloodstream infections. In this regard, new imaging tools employing specific tracers for molecular imaging [130,131,132,133] may be useful in the future to enable earlier and more accurate identification of endocardial involvement. Moreover, emerging approaches are exploring the combination of this technology with therapeutic modalities for simultaneous imaging and treatment [134].

### 7.3. Antimicrobial Strategies and Surgical Managment

As previously mentioned, few data are available regarding the best therapeutic approach to NHGNIE. Several important clinical questions remain unanswered:

- What should be considered as the first-line treatment of choice?
- Is there a role for combination therapy (especially in selected populations, e.g., patients with PVE or *P.* infections)?
- What is the most appropriate treatment duration?
- Is there a role for transition to oral therapy?
- Should patients with PVE not undergoing surgery receive suppressive antimicrobial treatment?

Little is also known regarding the best approach for difficult-to-treat pathogens, although a few case reports and case series have reported successful use of novel agents in this context [91,135,136,137,138,139,140,141].

Another critical issue in NHGNIE management is surgical timing, which is particularly relevant given that most patients are elderly, have multiple comorbidities, indwelling cardiac devices, or are at high risk of relapse (such as PWID) [142,143,144]. However, given the low proportion of NHGNIE cases and the inherent difficulty in conducting randomized controlled trials to evaluate surgical management, a definitive answer on this issue is unlikely. Management should therefore always be optimized within a multidisciplinary framework, the so-called “endocarditis team”, to tailor surgical decisions to each patient’s characteristics [145].

### 7.4. Translational Science and Novel Therapeutic Adjuncts

The mechanisms of adherence and biofilm formation are crucial for IE pathogenesis. While these phenomena have been extensively studied for Gram-positive pathogens, especially *S. aureus*, much less is known about Gram-negative bacteria, which also appears to exhibit remarkable interspecies variability. Further insight into NHGNIE pathogenesis is needed to inform potential preventive measures (such as colonization- and biofilm-resistant devices [146,147,148]) and novel therapeutic approaches, such as antibiofilm compounds [149].

Another promising avenue in the treatment of bacterial infections is the use of bacteriophages, which are viruses that are able to specifically and selectively infect and kill bacteria [150,151]. This approach may be particularly intriguing in IE, as some data suggest a potential antibiofilm effect of phages [152,153,154,155]. While pre-clinical data appear promising [156,157], evidence for their role in NHGNIE is extremely limited [158]. Further studies are needed to assess feasibility, identify barriers, and develop strategies to overcome potential challenges associated with this approach [159].

## 8. Conclusions

NHGNIE remains a relatively rare but clinically important form of IE, with epidemiologic patterns shaped by injection drug use, comorbidity burden, and healthcare-associated risk factors. Its pathogenesis is closely linked to biofilm formation and antimicrobial resistance, which complicate management and contribute to high morbidity and mortality. Although diagnostic advances (particularly molecular and imaging techniques) have improved case recognition, optimal treatment strategies remain uncertain, with conflicting data on the role of combination therapy and surgical intervention. Future progress will require multicenter prospective studies, integration of novel antimicrobial and adjunctive therapies, and a more refined understanding of pathogen-specific features. Until then, individualized patient-centered care within a multidisciplinary “endocarditis team” should serve as the cornerstone of effective NHGNIE management.

## Figures and Tables

**Table 1:** Epidemiological characteristics of selected cohorts of patients with NHGNIE.

Study	Year	Country, Setting	Cases	Median Age	Median Charlson	Previous IE	Nosocomial or Healthcare-Associated Acquisition	PWID	Source of Infection	Right-Sided IE	PVE
International											
Morpeth et al. 2007 [1]	2000–2005	International, multicenter	49/2761 (1.8%)	63		8%	Nosocomial 39% Healthcare-associated 17%	4%	Genitourinary 22% Gastrointestinal 13% Skin 9%	12%	59%
North America											
Shah et al. 2023 [2]	2010–2021	USA, multicenter	123	49	1	23%		52%		23%	17%
Lorenz et al. 2021 [3]	2011–2019	USA, monocenter	60/1036 (5.8%)	50	3			40%	IVDU-related 35% Gastrointestinal 18% Genitourinary 17% CVC 5%		25%
Veve et al. 2020 [4]	2011–2019	USA, monocenter	43	40		67%	Healthcare-associated 49%	93%		63%	30%
South America											
de Sousa et al. 2023 [5]	2006–2019	Brazil, multicenter	38/1154 (3.3%)	57		8%	Nosocomial 53% Healthcare-associated 26%	0%	CVC 29% Genitourinary 5% Gastrointestinal 5%	11%	47%
Burgos et al. 2019 [6]	1998–2016	Argentina, monocenter	31/452 (6.9%)	72			Healthcare-associated 70%	0%			46%
Europe											
Dorfler et al. 2025 [7]	2015–2021	Germany, monocenter	19/1093 (1.7%)	69	4	15%	Nosocomial 42%	0%	Genitourinary 50% CVC 31% Gastrointestinal 25%	0%	37%
Al Janabi et al. 2025 [8]	2008–2023	Sweden, multicenter	114/7426 (1.5%)	69		16%	Nosocomial 9% Healthcare-associated 5%	20%		9%	21%
Sebillotte et al. 2023 [9]	2007–2020	France, multicenter	77/3230 (2.4%)	69	2	8%	Healthcare-associated 36%	8%	Genitourinary 33% Gastrointestinal 17% Skin 13%	8%	23%
Loubet et al. 2015 [10]	2009–2014	France, monocenter	12 (4%)	51		33%	Healthcare-associated 33%	17%	Genitourinary 25% Gastrointestinal 25%	33%	67%
Calderón-Parra et al. 2021 [11]	2008–2018	Spain, multicenter	104/3910 (2.6%)	71	5		Nosocomial 48% Healthcare-associated 5%	4%	Venous catheter 26% Genitourinary 22% Gastrointestinal 6%	12%	33%
Noureddine et al. 2011 [12]	1984–2008	Spain, multicenter	24/961 (2.5%)	63	4		Nosocomial 29%	0%		4%	17%
Ertugrul Mercan et al. 2019 [13]	2007–2016	Turkey, multicenter	26	53		4%		0%		19%	19%
Falcone et al. 2018 [14]	2004–2011	Italy, multicenter	58/1722 (3.4%)	70			Healthcare-associated 41% Nosocomial 3%	9%	Genitourinary 28% Skin 14% Gastrointestinal 16%		28%
Asia											
Arora et al. 2021 [15]	2010–2020	India, monocenter	11/199 (5.5%)					55%		45%	18%
Thomas et al. 2020 [16]	2006–2016	India, monocenter	27/256 (10.7%)	49		35%					
Tran et al. 2017 [17]	2005–2014	Vietnam, monocenter	6/189 (3.2%)							17%	33%

NHGNIE—Non-HACEK Gram-Negative Infective Endocarditis; IE—Infective Endocarditis; PWID—people who inject drugs; PVE—prosthetic valve endocarditis; IVDU—intravenous drug Use; CVC—central venous catheter.

**Table 3:** Clinical characteristics and outcomes of patients with NHGNIE from selected cohorts.

Study	Fever	Complications	Vegetations	Heart Failure	Cardiac Abscesses	Embolic Events	Septic Shock	Recurrence/Relapse	Mortality
Dorfler et al. 2025 [7]		74%			32%	32%	11%		In hospital 21% 1 year 44%
Al Janabi et al. 2025 [8]						31%			In hospital 17%
Shah et al. 2023 [2]	61%		>10 mm 60%	34%		66%		9 months 15%	In hospital 14% 90 days 20%
de Sousa et al. 2023 [5]	58%		Median size 11 mm	47%	3%	55%			50%
Sebillotte et al. 2023 [9]	88%			16%	5%	31%	20%	5%	1 year 36%
Arora et al. 2021 [15]									In hospital 36%
Calderón-Parra et al. 2021 [11]	76%		69%	33%	17%	20%	21%	1%	In hospital 37% 1 year 42%
Lorenz et al. 2021 [3]			72% >10 mm 38%			42%			In hospital 6% 60 days 20%
Thomas et al. 2020 [16]	100%					51%			In hospital 30%
Veve et al. 2020 [4]						65%	40%		In hospital 5% 1 year 30%
Burgos et al. 2019 [6]	84%	21%		19%	3%	10%			In hospital 21%
Ertugrul Mercan et al. 2019 [13]	77%		88% Median size 11 mm	38%		23%			In hospital 23%
Falcone et al. 2018 [14]			86% Median size 14 mm	26%	5%	24%	3%		In hospital 14% 1 year 31%
Loubet et al. 2015 [10]	92%	58%	92%	8%	42%	58%		8%	In hospital 0% 1 year 8%
Noureddine et al. 2011 [12]		91%	92%	46%		50%	20%	0%	In hospital 41%
Morpeth et al. 2007 [1]	92%		80%	37%	25%	33%			In hospital 24%

**Table 4:** NGHNIE recommendations from available guidelines.

	European Society of Cardiology Guidelines [79]	American Heart Association Guidelines [99]
Antibiotic therapy	β-lactams in combination with aminoglycoside, sometimes with additional fluoroquinolones or cotrimoxazole.	Combination antibiotic therapy with β-lactams and either aminoglycosides or fluoroquinolones.
Duration of antibiotic therapy	6 weeks	6 weeks
Surgical therapy	Always recommended, as early as possible.	Cardiac surgery is reasonable for most patients, particularly when left-side valves are involved.
Other recommendations	Consultation with endocarditis team when available. In vitro bactericidal tests and monitoring of serum antibiotic concentrations.	Consultation with infectious diseases specialist is recommended. In vitro susceptibility testing.

**Table 5:** Studies evaluating combination therapy vs. monotherapy.

Study	Number of Patients	Pathogen	Combination Therapy	Therapy	Outcomes of Combination vs. Monotherapy
Shah et al. 2023 [2]	123	*Serratia marcescens* 41% *Pseudomonas aeruginosa* 21% *Escherichia coli* 10% *Klebsiella pneumoniae* 9%	43%	BL + AG 45% BL + FQ 42% Other 13%	No overall benefit of combination therapy *Serratia* and *Enterobacterales*: Clinical failure 10% vs. 26% (*p* = 0.09) 90-day mortality 15% vs. 41% (*p* = 0.037) *Pseudomonas aeruginosa*: Clinical failure 37% vs. 0% (*p* = 0.134) 90-day mortality 21% vs. 0%
Calderon-Parra et al. 2021 [11]	104	*Escherichia coli* 38% *Pseudomonas aeruginosa* 30% *Serratia* spp. 8% *Klebsiella* spp. 7%	79%	BL + AG 45% BL + FQ 30% Other 25%	In-hospital mortality (BL + FQ) aOR 0.29, CI 0.09–0.96
Lorenz et al. 2021 [3]	60	*Pseudomonas aeruginosa* 37% *Escherichia coli* 17% *Serratia* spp. 12% *Klebsiella pneumoniae* 10%	43%	BL + AG 38% BL + FQ 58% Other 4%	Composite outcome (60-day mortality/readmission/recurrence) aOR 0.45 (CI 0.13–1.6) Bacteremia recurrence 19% vs. 9% (*p* = 0.28) Readmission 31% vs. 41% (*p* = 0.36) Mortality 19% vs. 21% (*p* = 0.9) Adverse events 19% vs. 0% (*p* = 0.012)
Veve et al. 2020 [4]	43	*Pseudomonas aeruginosa* 68% *Serratia marcescens* 20% *Klebsiella* spp. 2%	76%	BL + AG 50% BL + FQ 34% Other 16%	No overall benefit of combination therapy BL + FQ: 90-day mortality/readmission aOR 6.7 (CI 1.4–30.4) 12-month readmission aOR 33.9 (CI 2.7–429.9)
Falcone et al. 2018 [14]	58	*Escherichia coli* 31% *Pseudomonas aeruginosa* 19% *Klebsiella pneumoniae* 10% *Serratia marcescens* 4%	86%	Non-carbapenem BL + AG 28% Non-carbapenem BL + FQ 16% Carbapenem + AG or FQ 8% Non-carbapenem BL + carbapenems ± AG or FQ 14%	Higher survival with penicillin/cephalosporin-based vs. carbapenem-based regimens (univariate analysis)
Morpeth et al. 2007 [1]	49	*Escherichia coli* 29% *Pseudomonas aeruginosa* 22% *Klebsiella* spp. 10% *Serratia* spp. 8%	63%	BL + AG 57% BL + FQ 30% BL + AG and FQ 7% Other 6%	In-hospital mortality 27% vs. 22% (*p* = 0.73)
Meena et al. 2024 [93]	218 (systematic review)	*Pseudomonas aeruginosa*	77%	Penicillin/cephalosporins + AG 54% Carbapenem + AG 13% FQ-based combination 15% Polymyxin-based combination 11% Other 7%	Mortality OR 0.64 (CI 0.34–1.20)
Shah et al. 2024 [91]	48	*Pseudomonas aeruginosa*	69%	BL + AG 61% BL + FQ 33% Other 6%	No benefit in response rate or survival Adverse events leading to discontinuation 21% vs. 0% (*p* = 0.08)
Walczak et al. 2023 [92]	15	*Pseudomonas aeruginosa*	60%	BL + AG 67% BL + FQ 33%	1-year mortality 36% vs. 75% (*p* = 0.282)
McCrary et al. 2025 [95]	159	*Serratia* spp.	43%	BL + AG 43% BL + FQ 54% Other 1%	In-hospital mortality aOR 0.15 (CI 0.03–0.74)
Shah et al. 2023 [96]	75	*Serratia* spp.	48%	BL + AG 33% BL + FQ 56% Other 11%	Clinical failure aOR 0.17 (CI 0.03–0.86) Microbiological failure 0% vs. 15% (*p* = 0.026) 90-day all-cause mortality 11% vs. 31% (*p* = 0.049) Adverse events leading to discontinuation 36% vs. 8% (*p* = 0.058)

BL—beta-lactams; AG—aminoglycosides; FQ: fluoroquinolones; *p*-value—*p*; aOR—Adjusted Odds Ratio; CI—Confidence Interval; OR—Odds Ratio.

**Table 6:** Studies evaluating surgery in NHGNIE.

Study	Number of Patients	Indication for Surgery	Surgery	Outcomes of Surgery vs. No Surgery
Dorfler et al. 2025 [7]	19	10 (53%)	8 (42%)	In-hospital mortality 25% vs. 18% (*p* = 1.0) 1-year mortality 25% vs. 45% (*p* = 0.633)
Shah et al. 2023 [2]	123	84 (68%)	49 (40%)	No surgery despite indication: Clinical failure aOR 7.44 (CI 2.37–23.4) 90-day mortality aOR 14.6 (CI 4.62–45.9)
De Sousa et al. 2023 [5]	38		8 (21%)	Mortality 42% vs. 58% (not significant)
Sebillotte et al. 2023 [9]	77		12 (16%)	No difference in 1-year mortality (*p* = 0.29)
Calderón Parra et al. 2021 [11]	104	64 (62%)	47 (45%)	No surgery despite indication: In-hospital mortality aOR 3.60 (CI 1.17–11.05)
Veve et al. 2020 [4]	43		10 (23%)	No difference in 90-day or 1-year mortality (*p* = 0.29)
Burgos et al. 2019 [6]	24		9 (38%)	No difference in in-hospital mortality
Ertugrul Mercan et al. 2019 [13]	26		10 (38%)	In-hospital mortality 20% vs. 25% (not significant)
Falcone et al. 2018 [14]	58		25 (43%)	Patients with complications (cardiac abscess, fistula, dehiscence, valve perforation, heart failure): mortality 18% vs. 60% (*p* = 0.049)
Morpeth et al. 2007 [1]	49		25 (51%)	In-hospital mortality 24% vs. 25% (*p* = 0.94)
McCrary et al. 2025 [95]	159 (*Serratia* spp.)		57 (36%)	Mortality aOR 0.14 (CI 0.03–0.64)
Shah et al. 2023 [96]	75 (*Serratia* spp.)	58 (77%)	34 (45%)	No surgery despite indication: Clinical failure aOR 3.84 (CI 4.5–105)
Shah et al. 2024 [91]	48 (*Pseudomonas* spp.)	30 (63%)	17 (35%)	No difference in clinical failure
Meena et al. 2024 [93]	218 (*Pseudomonas* spp, systematic review)		125 (57%)	Mortality aOR 0.41 (CI 0.20–0.82)

*p*-value—*p*; aOR—Adjusted Odds Ratio; CI—Confidence Interval; OR—Odds Ratio.

## Data Availability

No new data were created or analyzed in this study.

## Supplementary Materials

The following supporting information can be downloaded at: https://www.mdpi.com/article/10.3390/antibiotics14100980/s1. Table S1: Microbial etiology of patients with NHGNIE from selected cohort.

## Abbreviations

The following abbreviations are used in this manuscript:

NHGNIE	Non-HACEK Gram-Negative Infective Endocarditis
IE	Infective Endocarditis
PWID	people who inject drugs
PVE	prosthetic valve endocarditis
MDR	multidrug-resistant
EPS	extracellular polymeric substances
QS	quorum sensing
ExPEC	Extraintestinal Pathogenic *Escherichia coli*
UTI(s)	urinary tract infection(s)
ESC	European Society of Cardiology
ISCVID	International Society for Cardiovascular Infectious Diseases
CIED	cardiovascular implantable electronic device
PET/CT	positron emission tomography/computed tomography
18F-FDG	Fluorine-18 Fluorodeoxyglucose (radiotracer used in PET scans)
WBC SPECT/CT	white blood cell single-photon emission-computed tomography/computed tomography
CT	computed tomography
BCNIE	blood-culture-negative infective endocarditis
PCR	polymerase chain reaction
ESBL(s)	extended-spectrum beta-lactamase(s)
NVE	native valve endocarditis
CNS	central nervous system
MRSA	methicillin-resistant *Staphylococcus aureus*
VRE	vancomycin-resistant Enterococcus
AHA	American Heart Association
BL	beta-lactams
AG	aminoglycosides
FQ	fluoroquinolones
*p*-value	*p*
aOR	Adjusted Odds Ratio
CI	Confidence Interval
IDU	injection drug use
