# Supplementary material for: Epidemiology, Pathogenesis, Clinical Features, and Management of Non-HACEK Gram-Negative Infective Endocarditis
Source: Antibiotics (Basel). 2025 Sep 29;14(10):980. doi: 10.3390/antibiotics14100980 (PMC12561054; doi:10.3390/antibiotics14100980)
Supplement: Supplementary file 1 [file antibiotics-14-00980-s001.zip › antibiotics-3868735-supplementary.pdf]

Table S1 - Microbial etiology of patients with NHGNIE from selected cohorts

Study	*Pseudomonas aeruginosa*	*Klebsiella pneumoniae*	*Escherichia coli*	*Serratia marcescens*	MDR
Dorfler et al. 2025[1]		26%	26%		5%
Al Janabi et al. 2025[2]	13%	7%	28%	5%	
Shah et al. 2023[3]	21%	9%	10%	41%	21%
de Sousa et al. 2023[4]	21%	21%*	5%	16%	21%
Sebillotte et al. 2023[5]	7%	16%*	43%	12%	10%
Arora et al. 2021[6]	64%	9%			
Calderón-Parra et al. 2021[7]	30%	6%*	38%	8%*	
Lorenz et al. 2021[8]	37%	10%	17%	12%*	27%
Thomas et al. 2020[9]	19%		37%		55%
Veve et al. 2020[10]	68%	2%*		20%	7%
Burgos et al. 2019[11]	21%	8%	25%	13%	
Ertugrul Mercan et al. 2019[12]	26%	19%	26%		27%
Falcone et al. 2018[13]	19%	10%	31%	4%	28%
Tran et al. 2017[14]	33%				
Loubet et al. 2015[15]	25%		33%	8%	
Noureddine et al. 2011[16]	17%	8%	25%	4%*	
Morpeth et al. 2007[17]	22%	10%*	29%	8%*	

*Includes spp.

1. Dörfler, J.; Grubitzsch, H.; Schneider-Reigbert, M.; Pasic, M.; Pfäfflin, F.; Stegemann, M.; Sander, L.E.; Kurth, F.; Lingscheid, T. Non-HACEK Gram-Negative Bacilli Infective Endocarditis: Data from a Retrospective German Cohort Study. *Infection* **2025**, *53*, 405–413.
2. Al Janabi, J.; El Noaimi, M.; Sunnerhagen, T.; Snygg-Martin, U.; Rasmussen, M. Infective Endocarditis Caused by Non-HACEK Gram-Negative Bacteria, a Registry-Based Comparative Study. *Open Forum Infect. Dis.* **2025**, *12*, ofaf085.
3. Shah, S.; Clarke, L.G.; Shields, R.K. Epidemiology and Clinical Outcomes of Non-HACEK Gram-Negative Infective Endocarditis. *Open Forum Infect. Dis.* **2023**, *10*, ofad052.
4. de Sousa, L.P.; Fortes, C.Q.; Damasco, P.V.; Barbosa, G.I.F.; Golebiovski, W.F.; Weksler, C.; Garrido, R.Q.; Siciliano, R.F.; Lamas, C. da C. Infective Endocarditis Due to Non-HACEK Gram-Negative Bacilli: Clinical Characteristics and Risk Factors from a Prospective Multicenter Brazilian Cohort. *Trop. Med. Infect. Dis.* **2023**, *8*, 283.
5. Sebillotte, M.; Boutoille, D.; Declerck, C.; Talarmin, J.-P.; Lemaignen, A.; Piau, C.; Revest, M.; Tattevin, P.; Gousseff, M.; Groupe d'Epidémiologie et Recherche en Infectiologie Clinique du Centre et de l'Ouest (GERICCO) Non-HACEK Gram-Negative Bacilli Endocarditis: A Multicentre Retrospective Case-Control Study. *Infect. Dis. (Lond.)* **2023**, *55*, 599–606.
6. Arora, N.; Panda, P.K.; Cr, P.; Uppal, L.; Saroch, A.; Angrup, A.; Sharma, N.; Sharma, Y.P.; Vijayvergiya, R.; Rohit, M.K.; et al. Changing Spectrum of Infective Endocarditis in India: An 11-Year Experience from an Academic Hospital in North India. *Indian Heart J.* **2021**, *73*, 711–717.
7. Calderón Parra, J.; De Castro-Campos, D.; Muñoz García, P.; Olmedo Samperio, M.; Marín Arriaza, M.; De Alarcón, A.; Gutierrez-Carretero, E.; Fariñas Alvarez, M.C.; Miró Meda, J.M.; Goneaga Sanchez, M.Á.; et al. Non-HACEK Gram Negative Bacilli Endocarditis: Analysis of a National Prospective Cohort. *Eur. J. Intern. Med.* **2021**, *92*, 71–78.
8. Lorenz, A.; Sobhanie, M.M.E.; Orzel, L.; Coe, K.; Wardlow, L. Clinical Outcomes of Combination versus Monotherapy for Gram Negative Non-HACEK Infective Endocarditis. *Diagn. Microbiol. Infect. Dis.* **2021**, *101*, 115504.
9. Thomas, V.V.; Mishra, A.K.; Jasmine, S.; Sathyendra, S. Gram-Negative Infective Endocarditis: A Retrospective Analysis of 10 Years Data on Clinical Spectrum, Risk Factor and Outcome. *Monaldi Arch. Chest Dis.* **2020**, *90*, doi:10.4081/monaldi.2020.1359.
10. Veve, M.P.; McCurry, E.D.; Cooksey, G.E.; Shorman, M.A. Epidemiology and Outcomes of Non-HACEK Infective Endocarditis in the Southeast United States. *PLoS One* **2020**, *15*, e0230199.

11. Burgos, L.M.; Oses, P.; Iribarren, A.C.; Pennini, M.; Merkt, M.; Vrancic, M.; Camporrotondo, M.; Ronderos, R.; Sucari, A.; Nacinovich, F. Endocarditis infecciosa por bacilos gram negativos no HACEK. Experiencia en un centro de alta complejidad de la República Argentina (1998-2016). *Rev. Argent. Microbiol.* **2019**, *51*, 136–139.
12. Ertugrul Mercan, M.; Arslan, F.; Ozyavuz Alp, S.; Atilla, A.; Seyman, D.; Guliyeva, G.; Kayaaslan, B.; Sari, S.; Mutay Suntur, B.; Isik, B.; et al. Non-HACEK Gram-Negative Bacillus Endocarditis. *Med. Mal. Infect.* **2019**, *49*, 616–620.
13. Falcone, M.; Tiseo, G.; Durante-Mangoni, E.; Ravasio, V.; Barbaro, F.; Ursi, M.P.; Pasticci, M.B.; Bassetti, M.; Grossi, P.; Venditti, M.; et al. Risk Factors and Outcomes of Endocarditis Due to Non-HACEK Gram-Negative Bacilli: Data from the Prospective Multicenter Italian Endocarditis Study Cohort. *Antimicrob. Agents Chemother.* **2018**, *62*, doi:10.1128/AAC.02208-17.
14. Tran, H.M.; Truong, V.T.; Ngo, T.M.N.; Bui, Q.P.V.; Nguyen, H.C.; Le, T.T.Q.; Mazur, W.; Chung, E.; Cafardi, J.M.; Pham, K.P.N.; et al. Microbiological Profile and Risk Factors for In-Hospital Mortality of Infective Endocarditis in Tertiary Care Hospitals of South Vietnam. *PLoS One* **2017**, *12*, e0189421.
15. Loubet, P.; Lescure, F.-X.; Lepage, L.; Kirsch, M.; Armand-Lefevre, L.; Bouadma, L.; Lariven, S.; Duval, X.; Yazdanpanah, Y.; Joly, V. Endocarditis Due to Gram-Negative Bacilli at a French Teaching Hospital over a 6-Year Period: Clinical Characteristics and Outcome. *Infect. Dis. (Lond.)* **2015**, *47*, 889–895.
16. Noureddine, M.; de la Torre, J.; Ivanova, R.; Martínez, F.J.; Lomas, J.M.; Plata, A.; Gálvez, J.; Reguera, J.M.; Ruiz, J.; Hidalgo, C.; et al. Endocarditis sobre válvulas izquierdas por bacilos gran negativos: epidemiología y características clínicas. *Enferm. Infecc. Microbiol. Clin.* **2011**, *29*, 276–281.
17. Morpeth, S.; Murdoch, D.; Cabell, C.H.; Karchmer, A.W.; Pappas, P.; Levine, D.; Nacinovich, F.; Tattevin, P.; Fernández-Hidalgo, N.; Dickerman, S.; et al. Non-HACEK Gram-Negative Bacillus Endocarditis. *Ann. Intern. Med.* **2007**, *147*, 829–835.
